# Avoidant/restrictive food intake disorder (ARFID) symptoms in gender diverse adults and their relation to autistic traits, ADHD traits, and sensory sensitivities

**DOI:** 10.1186/s40337-025-01215-z

**Published:** 2025-02-17

**Authors:** Kai S. Thomas, Jennifer Keating, Avalon A. Ross, Kate Cooper, Catherine R. G. Jones

**Affiliations:** 1 School of Psychology, Cardiff University, Cardiff, Wales, UK; 2 Wales Institute of Social and Economic Research and Data, School of Social Sciences, Cardiff University, Cardiff, Wales, UK; 3 Department of Clinical, Educational and Health Psychology, University College London, London, England, UK; 4 Centre for Applied Autism Research, University of Bath, Bath, England, UK

**Keywords:** Avoidant/restrictive food intake disorder (ARFID), Gender diversity, Transgender, Autistic traits, ADHD traits, Sensory sensitivity

## Abstract

**Background:**

There is emerging evidence to suggest gender diverse people are overrepresented in avoidant/restrictive food intake disorder (ARFID) samples. However, the mechanisms underlying elevated risk for ARFID in this group are currently unknown. Gender diversity and neurodivergence commonly co-occur, with elevated sensory sensitivities reported to be a shared experience common across autism, attention deficit hyperactivity disorder (ADHD), and ARFID. We aimed to examine the unique contribution of sensory sensitivities, including hypo- and hyper-sensitivity, in predicting ARFID symptoms in gender diverse adults, whilst controlling for autistic and ADHD traits.

**Methods:**

Gender diverse adults (*N* = 182; 142 assigned female at birth; *M* age = 28.6 years) in the UK participated in an online survey. We examined correlations between their self-reported ARFID symptoms, sensory sensitivities, autistic traits (not including sensory sensitivities), and ADHD traits whilst controlling for weight and shape concerns. We then used hierarchical multiple regression to investigate the unique contribution of sensory sensitivities to ARFID symptoms whilst controlling for the other neurodivergent traits.

**Results:**

In our gender diverse sample, higher levels of ARFID symptoms were associated with higher levels of sensory sensitivities, autistic traits, and ADHD traits, after controlling for weight and shape concerns. Furthermore, sensory sensitivities, specifically hyper-sensitivity, uniquely predicted levels of ARFID symptoms once we accounted for autistic and ADHD traits.

**Conclusions:**

When considering neurodivergence, sensory hyper-sensitivities may be particularly relevant to ARFID symptomatology in gender diverse adults. Future research should explore associations between ARFID presentations and sensory sensitivities in large samples of gender diverse adults, to enable separate analyses by gender identity.

**Supplementary Information:**

The online version contains supplementary material available at 10.1186/s40337-025-01215-z.

## Abstract (plain language summary)

Gender diverse people, whose gender identity does not align with their sex assigned at birth, are at increased risk for eating disorders such as avoidant/restrictive food intake disorder (ARFID). Sensory sensitivities, autism and attention deficit hyperactivity disorder (ADHD) may increase a person’s risk of developing ARFID. Gender diverse people commonly report co-occurring autistic and ADHD traits, making it challenging to determine whether ARFID symptoms are linked to the autistic and ADHD traits they report, or whether ARFID symptoms are specifically associated with sensory sensitivities. We asked 182 gender diverse adults to complete an online questionnaire about their levels of ARFID symptoms, autistic traits, ADHD traits, and sensory sensitivities. We found gender diverse people with higher levels of ARFID symptoms also reported higher levels of autistic traits, ADHD traits, and sensory sensitivities. When we accounted for autistic and ADHD traits in our gender diverse sample, sensory hyper-sensitivity (i.e., an over-response to sensory information) had the strongest association with ARFID symptoms. Our findings suggest sensory sensitivities may be important to the development of ARFID symptoms in gender diverse people, over and above the influence of autistic and ADHD traits.

**Supplementary Information:**

The online version contains supplementary material available at 10.1186/s40337-025-01215-z.

## Introduction

Gender diverse people, whose gender identity does not align with their sex assigned at birth, are at elevated risk of developing eating disorders [1–10]. Eating disorders are prevalent across the gender diverse spectrum, including in binary trans individuals, such as trans men and trans women, and individuals who identify with an identity between or outside of the gender binary, such as non-binary, gender fluid, or genderqueer [11–13]. A recent meta-analysis reported estimated prevalence rates of eating disorders to be 17.7% in binary trans individuals [14]. There is some evidence to suggest these rates may be even higher for individuals who identify with a non-binary identity [13]. These estimated rates are considerably elevated compared to rates of 1.8% and 0.2% reported in cisgender women and men, respectively [15]. Eating disorder symptomatology in gender diverse people is likely to serve a number of functions, such as attaining body characteristics that are more aligned with their gender identity [2, 4, 7], controlling pubertal development, such as menstruation cessation for individuals assigned female at birth [6, 11, 16, 17], and/or managing heightened levels of emotional distress due to interpersonal and intrapersonal stressors [18, 19]. The majority of research with gender diverse people to-date has focused on eating pathology related to weight and shape concerns [1, 11, 20, 21], with little attention given to other types of feeding and eating disorders.

Avoidant/restrictive food intake disorder (ARFID) is a feeding and eating disorder characterised by an inadequate intake of nutrition and/or energy that is associated with significant physical and/or psychological difficulties, such as nutritional deficiencies, weight loss, and a marked interference with psychosocial functioning [22]. ARFID was introduced in the Diagnostic and Statistical Manual of Mental Disorders, fifth edition (DSM-5; 22) in 2013, where it replaced Feeding Disorder of Infancy and Early Childhood, recognising that ARFID symptoms can occur at any age. ARFID is distinguished from other eating disorders by an absence of weight or shape concerns [22]. Restrictive and avoidant eating behaviour presented in ARFID can be related to aversions to sensory properties (i.e., related to the texture, taste, smell, or appearance of the food), low appetite or limited interest in eating, and/or fear of negative consequences from eating (e.g., choking; 23). According to the three-dimensional model of ARFID proposed by Thomas et al. [23], these underpinning drivers are not mutually exclusive and can vary in severity, resulting in heterogeneous presentations of ARFID.

The true prevalence of ARFID is currently unknown and estimates have primarily been focussed on child samples. Prevalence estimates vary from 1.5 to 64% among clinical eating disorder populations and < 1–15.5% in non-clinical cohorts [24]. Two of the only studies to examine ARFID prevalence in older adolescents and adults from the general population reported rates of 0.3% and 4.7%, assessing ARFID using interviews and self-report questionnaires, respectively [25, 26]. Research examining the developmental trajectories of ARFID is lacking, but there is some evidence to suggest that ARFID typically presents before adulthood and can remain stable across the lifespan [27]. Therefore, ARFID symptomatology is not confined to childhood, and further research focussed specifically on adults is necessary to increase understanding of ARFID symptoms across the lifespan [24, 28, 29].

There is emerging evidence to suggest gender diverse people are overrepresented in ARFID samples. In a retrospective chart review of adults treated for ARFID at an eating disorder clinic, MacDonald, Liebman [30] found 16.7% of their sample identified as transgender, non-binary, or another gender diverse identity. This figure far exceeds the prevalence that would be expected, given gender diverse people make up less than 2% of the general population [31, 32]. The only other studies exploring ARFID in gender diverse people have focussed on screening for ARFID using self-report measures [33–35]. All three studies used the Nine-Item ARFID Screen (NIAS; 36), a well-established screening measure for ARFID. The NIAS has three subscales that capture symptoms associated with the three main overlapping ARFID presentations: sensory sensitivity, lack of interest, and fear of aversive consequences [36]. The measure has recently been validated in gender diverse youth and young adults attending a US gender clinic [35]. In this study, Zickgraf, Garwood [35] reported 22% of their sample screened positive for ARFID, with gender diverse people assigned female at birth scoring significantly higher on the NIAS compared to those assigned male at birth. Despite growing evidence suggesting a link between ARFID and gender diversity, the underlying mechanisms are currently unclear.

Neurodivergence may play a significant role in the co-occurrence of gender diversity and ARFID. Research has found an overlap between autism, attention deficit hyperactivity disorder (ADHD), and gender diversity [37–41]. Both autism and ADHD are around 2–6 times more common in gender diverse individuals compared to cisgender individuals, whose gender identity aligns with their sex assigned at birth [41]. In addition, both autistic and non-autistic gender diverse people endorse more autistic traits on self-report measures compared to cisgender people [41–45]. Autistic people and people with ADHD are found to be at elevated risk of developing ARFID [30, 46]. For example, in a cohort of adults attending treatment for ARFID, MacDonald, Liebman [30] found 16.7% of the sample had an autism diagnosis whilst 26.2% had an ADHD diagnosis. Previous research has not yet explored the relations between autistic traits, ADHD traits, and ARFID symptoms in a gender diverse sample.

Based on the three maintaining mechanisms typically seen in ARFID [23, 24], autistic people and people with ADHD more commonly exhibit presentations driven by food-related sensory sensitivities and/or a lack of appetite/interest in eating, compared to those driven by a fear of aversive consequences of eating [30, 46]. The heightened occurrence of these ARFID presentations may be reflective of behavioural and cognitive patterns characteristic of and/or associated with both autism and ADHD. These include sensory sensitivities, encompassing both hyper- and hypo-sensitivity, emotion regulation differences, hyperactivity and impulsivity, and behavioural and cognitive rigidity, such as stimming, monotropic interests, and difficulties coping with novelty or uncertainty [47–55]. These characteristics can lead to eating behaviours typically seen in ARFID, such as rigid rules around food preparation, a limited food repertoire, insistence on specific brands of food only, as well as increased consumption of foods with pleasant sensory properties. Taken together, these findings suggest the elevated rates of ARFID in gender diverse people may be driven by the high rates of co-occurring neurodivergence.

When considering neurodivergent traits that may be relevant to ARFID, sensory sensitivities may be particularly relevant as a transdiagnostic feature. They are included in the diagnostic criteria for both autism and ARFID [22] and are also observed at elevated levels in people with ADHD [53], suggesting they are a transdiagnostic experience. Sensory sensitivities are also present in samples independent of neurodivergent traits, such as individuals with migraine [56], anxiety and depressive disorders [57], and the entire spectrum of eating disorders [58, 59]. Sensory hyper-sensitivities, defined as an over-response (i.e., speed, intensity, or duration of response) to sensory stimuli [60], and sensory hypo-sensitivities, defined as an under-response to sensory stimuli [61], have both been reported in autistic children and adults [62–64], as well as children and adults with ADHD [65–67].

Although limited, there is evidence to suggest both autistic and non-autistic gender diverse people experience elevated levels of sensory sensitivities compared to cisgender autistic and non-autistic people, respectively [41]. These sensory sensitivities may contribute to increased gender dysphoria and elevated distress resulting in a heightened risk of developing ARFID symptoms. Qualitative research with autistic gender diverse adults suggests sensory sensitivities may exacerbate feelings of gender dysphoria by increasing sensitivity to negative experiences of one’s body, known as ‘sensory dysphoria’ [63]. These sensory experiences can include facial hair growth, menstruation, and wearing clothing that is particularly uncomfortable due to the fabric or shape [63]. It is possible that sensory sensitivities are a key driver of ARFID symptoms in gender diverse people, alongside co-occurring neurodivergent traits.

In the current study, we aimed to examine the unique contribution of sensory sensitivities to levels of ARFID symptoms in gender diverse adults, whilst controlling for autistic traits (not including sensory sensitivities) and ADHD traits. Given that commonly used self-report measures of ARFID, such as the NIAS, are not able to reliably differentiate between restrictive eating behaviours specific to ARFID and those related to other eating disorders [68], we also controlled for weight and shape concerns. We hypothesised that: (1) ARFID symptoms would be positively correlated with levels of autistic traits, ADHD traits, and sensory sensitivities after controlling for weight and shape concerns and (2) sensory sensitivities would uniquely contribute to levels of ARFID symptoms in our gender diverse sample.

## Methods

### Participants

A total of 281 participants consented to participate in the study. All participants were recruited through social media advertisements and relevant support organisations. Advertisements specified that the aims of the research were to understand how eating behaviours were related to gender identity and neurodivergent characteristics. Participants were required to identify as gender diverse, be aged 18 years or older, be currently based in the UK, and be fluent in English. Eighty participants were excluded due to incomplete responses (> 10% missing items on individual questionnaires), one participant was excluded due to failure of attention checks, and 18 participants were excluded as they reported identifying as cisgender or did not provide their gender identity. This resulted in a final sample of 182 participants (*M* age = 28.60 years, *SD* = 8.57, ranged from 18 to 70 years). An a priori power analysis performed using G*Power indicated a minimum sample size of 178 was needed for a linear multiple regression with eleven predictors, assuming a medium effect size (*f*^2^ = 0.15), alpha of 0.05, and power of 0.95 [69].

The project received ethical approval from the University’s School of Psychology Ethics Committee (EC.23.10.10.6846GA3). Informed consent was obtained online from all participants before completing the questionnaires. Demographic information and diagnostic history are presented in Table 1, with additional demographics (ethnicity, highest level of education and sexual orientation) included in the Supplementary Materials (Table S1).

**Table 1:** Sample characteristics (*N* = 182)

	Frequency	% of the sample
**Sex assigned at birth**		
Female	142	78.0
Male	31	17.0
Prefer not to say/missing	9	4.9
**Gender identity** ^a^		
Trans man	44	24.2
Trans woman	21	11.5
Gender expansive^b^	28	15.4
Non-binary^b^	64	35.2
Trans masculine non-binary^b^	18	9.9
Trans feminine non-binary^b^	7	3.8
**Eating disorder diagnosis** ^c^		
Yes	28	15.4
Anorexia Nervosa	15	
Avoidant/Restrictive Food Intake Disorder	3	
Bulimia Nervosa	2	
Eating Disorder Not Otherwise Specified	8	
No	153	84.1
Prefer not to say	1	0.5
**Status of Eating Disorder**		
Current	19	10.4
Historical	8	4.4
Unsure	1	0.5
**Autism diagnosis** ^c^		
Yes	54	29.7
No	126	69.2
Prefer not to say	2	1.1
**ADHD diagnosis** ^c^		
Yes	47	25.8
No	131	72.0
Prefer not to say	4	2.2
**Mental health diagnoses** ^c^		
Yes	135	74.2
Depressive Disorders	111	
Anxiety Disorders	93	
Post-Traumatic Stress Disorder	27	
Obsessive-Compulsive Disorder	16	
Borderline Personality Disorder	11	
No	46	25.3
Prefer not to say	1	0.5

Note.^a^To verify that participants identified as gender diverse, they were asked to indicate whether their gender identity aligned with their sex assigned at birth. They were then asked to specify their gender identity using an open-text box. These free-text responses were coded by the authors into one of the six categories displayed

^b^Sex assigned at birth for participants in each of these categories: Gender expansive = 2 people assigned male at birth (AMAB), 23 people assigned female at birth (AFAB); Non-binary = 7 people AMAB, 55 people AFAB; Transmasculine non-binary = 1 person AMAB, 17 people AFAB; Transfeminine non-binary = 5 people AMAB, 1 person AFAB

^c^Participants were asked whether they had received a formal diagnosis of an eating disorder of any type, autism, ADHD, or diagnosis of a mental health condition. If participants responded ‘Yes’ to either an eating disorder or mental health diagnosis, they were given the option to specify one or more diagnoses using a free text response

*N* = 24 (13.2% of the sample) reported both autism and ADHD diagnoses

### Procedure

The study was hosted on an online platform [70]. Participants first answered a screening question to allow us to identify invalid low-quality responses [71]. In this screening question, participants were required to answer an identification question involving processing, understanding, and responding. Participants were presented with a snow sculpture and asked to briefly describe what they predicted would happen to this sculpture on a warm, sunny day. We screened individual free-text responses from each participant to ensure these were consistent with a description of melting snow. Participants were then asked to provide demographic information and complete a series of self-report questionnaires examining eating disorder behaviours and cognitions, sensory sensitivities, and neurodivergent traits. An additional measure of internalising symptoms was also included but was not relevant to the current research questions. An attention check was presented twice during the series of questionnaires, at the start and half-way through completion. This read: “Please ignore the question below and select both ‘yes’ and ‘unsure’. This way, we can be more confident that you are reading the questions carefully and will pay attention throughout the study. Do you frequently get less than 7 hours of sleep?”. Response options were ‘Yes’, ‘No’, or ‘Unsure’. After completing the questionnaires, participants were offered the option to enter a prize draw to win a monetary voucher as compensation for their time. Participants’ contact details were collected via a separate link to their data.

### Measures

#### Nine-item ARFID screen (NIAS; 36)

The NIAS is a nine-item self-report measure of ARFID symptoms. It is comprised of three subscales: the picky/selective eating subscale measuring sensory aversion to food (e.g., “I dislike most foods that other people eat”), the appetite subscale measures a lack of interest in food or eating (e.g., “I am not very interested in eating; I seem to have a smaller appetite than other people”), and the fear subscale (e.g., “I eat small portions because I am afraid of GI discomfort, choking, or vomiting”). Participants respond on a 6-point scale from 0 (Strongly disagree) to 5 (Strongly agree). The three subscales (ranging from 0 to 15) can be summed to create a total score (ranging from 0 to 45). Higher scores indicate higher levels of ARFID symptoms. The NIAS has recently been validated in gender diverse youth and young adults attending a US gender clinic [35]. In the current study, we only used the NIAS total score in our analyses (Cronbach’s α = 0.866).

#### Autism spectrum quotient short (AQ-S)

The AQ-S [72] is an abridged version of the AQ [73], a 50-item self-report measure of autistic traits in adults. The AQ-S contains a subset of 28 items from the AQ which form five subscales: social skills, routine, switching, imagination, and numbers/patterns. Participants respond on a 4-point scale from 1 (Definitely agree) to 4 (Definitely disagree) according to their endorsement of each statement. The scoring is then reversed for items where an ‘agree’ response is characteristic of autism (e.g., ‘I frequently get strongly absorbed in one thing’). Higher scores indicate higher levels of autistic traits. Items are summed to calculate scores, including the total score which ranges from 28 to 112. Subscales ranged from 8 to 32 (Social skills and Imagination), 4–16 (Routine and Switching), or 5–20 (Numbers/patterns), depending on their number of items. Hoekstra, Vinkhuyzen [72] recommended including one item (‘New situations make me anxious’) in the scoring of both the social skills and routine subscales for English samples. As our sample were all based in the UK and fluent in English, we followed this recommendation. Cronbach’s α for the AQ-S were as follows: total score = 0.866; social skills subscale score = 0.795; routine subscale score = 0.645; switching subscale score = 0.563; imagination subscale score = 0.786; numbers and patterns subscale score = 0.784. We used all five subscales in our analyses.

#### Adult ADHD self-report scale (ASRS)

The ASRS [74] is an 18-item self-report measure of ADHD traits. For each item (e.g., how often do you have problems remembering appointments or obligations?), the participant is asked to respond on a 5-point Likert scale (‘Never’ to ‘Very often’), based on how they have felt/behaved over the past 6 months. Depending on the item, responses are either scored as 0 or 1. The total score is calculated through summing the items and ranges from 0 to 18. Higher scores indicate higher levels of current ADHD traits. Three subscales can be calculated from the items based on the factors identified by Stanton, Forbes [75]: inattentive (ranges from 0 to 9), hyperactive/impulsive motor (0–5) and hyperactive/impulsive verbal (0–4). Cronbach’s α for the ASRS was as follows: total score = 0.848, inattentive subscale score = 0.754, hyperactive/impulsive motor subscale score = 0.698, hyperactive/impulsive verbal subscale score = 0.695. We used all three subscales in our analyses.

#### Glasgow sensory questionnaire (GSQ)

The GSQ [76] is a self-report measure of sensory sensitivities across seven sensory domains (visual; auditory; gustatory; olfactory; tactile; vestibular and proprioceptive). The 42 items are evenly split to measure hyper- and hypo-sensitivity. The measure was initially constructed based on sensory sensitivities associated with autism, but has been found to be reliable in both autistic and non-autistic adults [77]. Participants respond based on the frequency of each type of sensory experience (e.g., Do you react very strongly when you hear an unexpected sound?) on a 5-point scale from 0 (Never) to 4 (Always). Given sensory sensitivities are a key component of ARFID, we removed overlapping eating-related items (items 2, 27, 23, 26, 28, and 40) from the GSQ before scoring. Hypo- and hyper-sensitivity subscales are calculated by summing corresponding items (using the adapted scoring method, scores ranged from 0 to 72), whilst total scores are calculated by summing all items (0-144). Higher scores indicate higher levels of sensory sensitivities. Cronbach’s α was calculated for total score (α = 0.923) and both hyper-sensitivity (α = 0.888) and hypo-sensitivity (α = 0.841) subscales. We used both hyper- and hypo-sensitivity subscales in our analyses.

#### Eating disorder examination questionnaire (EDE-Q)

The EDE-Q [78] assesses eating disorder behaviours and cognitions over the previous 28 days (e.g., on how many of the past 28 days have you had a definite fear that you might gain weight? ), with responses captured on a 7-point scale ranging from 0 (‘no days’) to 6 (‘every day’). Averaging the scores of relevant items is used to capture four subscales: restraint, eating concern, shape concern, and weight concern. These subscale scores are averaged to create a global EDE-Q score (ranging from 0 to 6), with higher scores indicating a greater endorsement of eating disorder symptoms. Only the shape concern and weight concern subscales were used in the current analyses. The EDE-Q is a well-established measure that has been validated in gender diverse people [12, 79, 80]. Cronbach’s α for the shape concern (α = 0.896) and weight concern (α = 0.838) subscales in the current study were acceptable.

### Statistical analyses

All statistical analyses were conducted using R Statistical Software (version 4.3.2; R Core Team, 2021), accessed through R Studio (version 23.09.1). Missing values were low across all measures (< 0.35% of all datapoints) and mean imputation was used to replace these. The mean, standard deviation, and range were calculated for each subscale of the NIAS, AQ-S, ASRS, and GSQ, in addition to the shape concern and weight concern subscales of the EDE-Q. To examine our first hypothesis that ARFID symptoms would be positively correlated with levels of autistic traits, ADHD traits, and sensory sensitivities, Spearman’s correlations were conducted between ARFID symptoms (NIAS total score) and the subscales of the AQ-S (autistic traits), ASRS (ADHD traits), and GSQ (sensory sensitivities). These associations were further examined using partial correlations to control for weight and shape concerns (EDE-Q). To address our second hypothesis that sensory sensitivities would uniquely contribute to levels of ARFID symptoms, multiple linear regressions were conducted to examine the contributions of each independent variable (autistic traits, ADHD traits, and sensory sensitivities) on ARFID symptoms. In the multiple linear regression model, our control variables, sex at birth and EDE-Q Shape and Weight Concern subscales, were entered at Step 1, followed by GSQ hyper- and hypo-sensitivity subscales at Step 2, and AS-Q and ASRS subscales at Step 3. For the linear regression model, there was independence of residuals, no evidence of multicollinearity, homoscedasticity was present, and the assumption of normality was met, as assessed by a P-P Plot. The regression model was repeated with individuals with and without an eating disorder diagnosis (current and/or historical). Lastly, we conducted descriptive statistics and exploratory correlation analyses for each gender diverse group (Supplementary Materials).

## Results

The mean, standard deviation, and range for each subscale of the NIAS, AQ-S, ASRS, and GSQ, in addition to the shape concern and weight concern subscales of the EDE-Q for the whole sample are presented in Table 2. Descriptive statistics for each gender identity category (trans masculine, trans feminine, non-binary, gender expansive) are presented in Supplementary Materials.

Our sample reported a moderate level of ARFID symptoms, with mean scores on each NIAS subscale below the proposed screening cutoffs of ≥ 10 (picky eating), ≥ 9 (appetite), and ≥ 10 (fear) by Burton Murray, Dreier [81]. Picky eating was the most endorsed presentation of ARFID across the sample, with 53 participants (29.1%) scoring above the NIAS-picky eating subscale cutoff. This was closely followed by the 49 participants (26.9%) who scored above the NIAS-appetite cutoff, while only 23 participants (12.6%) met the cutoff score for the NIAS-fear subscale. Autistic traits were elevated in our sample, with 88.5% of the overall sample exceeding the screening cutoff score (> 65) suggested by Hoekstra, Vinkhuyzen [72]. Eating concern and weight concern mean scores, measured using the EDE-Q, were slightly higher than previously reported in both binary transgender [80] and gender-expansive populations [12].

**Table 2:** Descriptive statistics for the questionnaires (*n* = 182)

	Min-Max	Available scores	Mean (SD)
**ARFID symptoms (NIAS)**	0–44	0–45	15.91 (10.49)
Picky eating	0–15	0–15	6.71 (4.85)
Appetite	0–15	0–15	5.69 (4.44)
Fear	0–15	0–15	3.50 (4.33)
**Autistic traits (AQ-S)**	38–107	28–112	80.02 (12.25)
Social skills	8–32	8–32	23.63 (4.53)
Routine	4–16	4–16	12.99 (2.26)
Switching	6–16	4–16	13.00 (2.25)
Imagination	8–32	8–32	19.81 (5.26)
Numbers and patterns	5–20	5–20	14.28 (3.54)
**ADHD traits (ASRS)**	1–18	0–18	12.36 (4.01)
Inattentive	0–8	0–9	6.04 (1.98)
Hyperactivity/impulsivity motor	0–5	0–5	3.41 (1.48)
Hyperactivity/impulsivity verbal	0–4	0–4	2.21 (1.35)
Sensory sensitivities (GSQ)*	12–115	0-144	69.28 (21.61)
Hyper-sensitivity	6–66	0–72	37.95 (12.50)
Hypo-sensitivity	5–55	0–72	31.33 (10.55)
**Shape concern (EDE-Q)**	0–6	0–6	3.54 (1.56)
**Weight concern (EDE-Q)**	0–6	0–6	3.12 (1.61)

Note. NIAS: Nine-Item ARFID Scale, ARFID: Avoidant/Restrictive Food Intake Disorder; AQ-S: Autism Spectrum Quotient - Short, ADHD: Attention-deficit Hyperactivity Disorder, ASRS: Adult ADHD Self-Report Scale, GSQ: Glasgow Sensory Questionnaire, EDE-Q: Eating Disorder Examination Questionnaire

*Adapted GSQ scales were used (removing any eating-related items)

In relation to the first hypothesis, correlational analyses revealed significant positive associations between ARFID symptoms (NIAS total score) and autistic traits (AQ-S), ADHD traits (ASRS), and sensory sensitivities (GSQ) (Table 3). The only associations that were not significant were between the switching subscale (AQ-S) and ARFID symptoms (NIAS total score) and the verbal subscale (ASRS) and ARFID symptoms (NIAS total score). Furthermore, there were significant positive correlations between all autistic and ADHD traits and both hyper- and hypo-sensory sensitivities.

Partial correlations were conducted with the variables above, while controlling for shape concern and weight concern subscales (EDE-Q) (Table 4). Similar significant correlations remained for all subscales; however, the correlation between the AQ-S switching subscale and NIAS total score was now also significant (*p* =.032). Correlations conducted for each of the gender diverse groups (see Supplementary Materials) were broadly consistent with the whole sample analyses. However, participants in the gender expansive group displayed additional weak positive correlations between ARFID symptoms and both sensory hypo-sensitivities and some autistic traits.

**Table 3:** Spearman’s correlations between neurodivergent trait subscales, sensory sensitivities, shape and weight concerns, and NIAS total score

	NIAS Total Score	GSQ Hyper-sensitivity	GSQ Hypo-sensitivity
**Autistic traits (AQ-S)**	0.405***	0.629***	0.399***
Social skills	0.282***	0.456***	0.331***
Routine	0.271***	0.406***	0.284***
Switching	0.129	0.324***	0.244***
Imagination	0.306***	0.362***	0.284***
Numbers and patterns	0.323***	0.538***	0.549***
**ADHD traits (ASRS)**	0.186*	0.395***	0.605***
Inattentive	0.163*	0.301***	0.420***
Hyperactivity/impulsivity motor	0.253***	0.430***	0.568***
Hyperactivity/impulsivity verbal	0.096	0.250***	0.383***
**Sensory sensitivities (GSQ)**			
Hyper-sensitivity	0.557***		
Hypo-sensitivity	0.403***		
**Shape concern (EDE-Q)**	0.100		
**Weight concern (EDE-Q)**	0.122		

*p <.05, **p <.01, ***p <.001.

Note: NIAS: Nine-Item ARFID Scale; ARFID: Avoidant/Restrictive Food Intake Disorder; AQ-S: Autism Spectrum Quotient– Short; ASRS: Adult ADHD Self-Report Scale; GSQ: Glasgow Sensory Questionnaire; EDE-Q: Eating Disorder Examination Questionnaire

**Table 4:** Partial correlations between neurodivergent trait subscales, sensory sensitivities and ARFID symptoms (NIAS) while controlling for EDE-Q weight and shape concern subscales

	NIAS Total Score
**Autistic traits (AQ-S)**	0.389***
Social skills	0.280***
Routine	0.284***
Switching	0.160*
Imagination	0.288***
Numbers and patterns	0.304***
**ADHD traits (ASRS)**	0.179*
Inattentive	0.169*
Hyperactivity/impulsivity motor	0.259***
Hyperactivity/impulsivity verbal	0.044
**Sensory sensitivities (GSQ)**	
Hyper-sensitivity	0.558***
Hypo-sensitivity	0.417***

*p <.05, **p <.01, ***p <.001

Note: NIAS: Nine-Item ARFID Scale; ARFID: Avoidant/Restrictive Food Intake Disorder; AQ-S: Autism Spectrum Quotient– Short; ASRS: Adult ADHD Self-Report Scale; GSQ: Glasgow Sensory Questionnaire; EDE-Q: Eating Disorder Examination Questionnaire

With regard to the second hypothesis, multiple linear regression analysis investigated the unique contribution of sensory sensitivities, autistic traits, and ADHD traits on ARFID symptoms (Table 5). Sex assigned at birth and shape and weight concerns (EDE-Q) were controlled for at Step 1 and this model was not statistically significant. The addition of the hyper-sensitivity and hypo-sensitivity subscales at Step 2 was statistically significant, with an increase in model fit of 0.304. In Step 3, autistic traits (AQ-S) and ADHD traits (ASRS) that were significant correlates of ARFID symptoms were added into the model, resulted in a small increase in model fit. In this final model, hyper-sensitivity was the only significant predictor of NIAS scores ($\:\beta\:$*= 0.499*, *p*<.001). Higher hyper-sensitivity scores were associated with higher scores on the NIAS. Additional exploratory analysis confirmed that the pattern of data were the same when weight and shape concerns were not controlled for in the model.

**Table 5:** Regression of sensory, attention, and autistic traits predicting ARFID scores

	NIAS Total Score
**Step 1**	
Sex assigned at birth	0.035
EDE-Q Shape concerns	− 0.151
EDE-Q Weight concerns	0.240
$\:{R}^{2}$	0.016
F	0.954
**Step 2**	
Sex assigned at birth	− 0.060
EDE-Q Shape concerns	− 0.051
EDE-Q Weight concerns	− 0.041
GSQ Hyper-sensitivity	0.575***
GSQ Hypo-sensitivity	0.022
$\:{R}^{2}$	0.320
F	16.450***
**Step 3**	
Sex assigned at birth	− 0.065
EDE-Q Shape concerns	− 0.023
EDE-Q Weight concerns	− 0.072
GSQ Hyper-sensitivity	0.499***
GSQ Hypo-sensitivity	0.034
AQ-S Social skills	0.019
AQ-S Routine	0.053
AQ-S Imagination	0.061
AQ-S Numbers and patterns	0.000
ASRS Inattentive	− 0.034
ASRS Hyperactivity/impulsivity motor	0.050
$\:{R}^{2}$	0.329
F	7.521***

*p <.05, **p <.01, ***p <.001

Note: NIAS: Nine-Item ARFID Scale; ARFID: Avoidant/Restrictive Food Intake Disorder; AQ-S: Autism Spectrum Quotient– Short; ASRS: Adult ADHD Self-Report Scale; GSQ: Glasgow Sensory Questionnaire; EDE-Q: Eating Disorder Examination Questionnaire

## Discussion

The present study is the first to examine the unique contribution of sensory sensitivities in predicting ARFID symptoms in a sample of gender diverse adults. Importantly, we controlled for autistic and ADHD traits, both of which are associated with sensory processing differences [62–67], as well as overrepresented in the gender diverse population [41, 63]. In line with our first hypothesis, we found higher levels of ARFID symptoms were associated with higher levels of sensory sensitivities, autistic traits, and ADHD traits after controlling for weight and shape concerns. In line with our second hypothesis, we found sensory hyper-sensitivity to be the only unique predictor of ARFID symptoms when controlling for autistic and ADHD traits. This pattern of findings suggests sensory hyper-sensitivities may be central to the experiences of ARFID symptomatology in gender diverse adults, over and above autistic and ADHD traits.

We found ARFID symptoms were positively correlated with autistic and ADHD traits, as well as sensory hyper- and hypo-sensitivities in gender diverse adults. These findings are consistent with research from cisgender populations, demonstrating a high co-occurrence between ARFID symptomatology and sensory sensitivities, autism, and ADHD [82–91]. Our findings suggest gender diverse people with higher levels of sensory sensitivities, autistic traits, and ADHD traits are at elevated risk for developing ARFID symptoms, which is broadly in line with findings from cisgender populations [88]. However, compared to cisgender samples using the same measures, gender diverse people in our study reported elevated levels of sensory sensitivities [76], ARFID symptoms [68], and neurodivergent traits [92, 93]. The level of ARFID symptoms in our sample was similar to previously published values for gender diverse youth and young adults [35] and autistic traits were closely aligned with previously published AQ-S subscale scores for autistic gender diverse adults [94]. In a recent study measuring sensory sensitivities in gender diverse adults, Warrier, Greenberg [41] found higher levels of sensory hypersensitivity in gender diverse individuals compared to cisgender individuals when using the Sensory Perception Quotient (SPQ; 95). Therefore, our study findings provide support for a body of literature demonstrating ARFID symptoms, sensory sensitivities and neurodivergent traits are elevated in gender diverse adults.

It is also important to highlight that weight and shape concern, which were controlled for in our analyses, were elevated in our gender diverse sample compared to previous literature with largely cisgender samples in the UK [93, 96]. These findings suggest gender diverse adults are likely to report a range of eating pathologies, but the individual’s specific pattern of neurodivergent or related characteristics may contribute to the type of eating pathology they experience. For example, ARFID symptoms may be particularly prevalent in individuals with higher levels of sensory sensitivities, compared to characteristics such as intense interests and preoccupations with food and weight, which are more likely to present with anorexia nervosa symptoms. Further, the trajectories of these symptoms and co-occurring factors may change across time. For example, sensory sensitivities may drive the initial development of restrictive eating behaviours, but symptoms may be later maintained by neurodivergent traits like cognitive rigidity, insistence on sameness, and intense interests [86]. Future research examining associations between ARFID, sensory sensitivities, and neurodivergent traits should also consider differences between individuals who develop ARFID in childhood compared to those with adult-onset ARFID. Research suggests individuals with adult-onset ARFID most commonly endorse the driver ‘fear of aversive consequences’ [27], which differs from the mixed presentations of ARFID reported in children [97].

In our correlational analysis, we explored the associations between ARFID symptoms and categories of autistic and ADHD traits. Both before and after controlling for weight and shape concerns, we found ARFID symptoms were positively associated with all autistic and ADHD characteristics captured in the measures except for verbal hyperactivity/impulsivity. Switching, captured in the measure of autistic traits, was not significantly associated with ARFID symptoms before controlling for weight and shape concerns. Our findings are consistent with research demonstrating individuals with ARFID display repetitive and routinised behaviours around food and eating [24, 86, 98], as well as differences in social responsiveness [90]. Indeed, the diagnostic criteria for ARFID can include difficulties in social functioning, such as inability to sustain social relationships and participate in social activities, as a consequence of these avoidant and restrictive eating behaviours [22]. Previous research has also reported associations between ARFID symptoms and rates of ADHD diagnoses/traits in children and adolescents [99, 100]. However, our study is the first to demonstrate this association in adults. Specifically, we found inattentiveness and hyperactivity/impulsivity to be positively associated with ARFID symptoms, although this association was no longer significant when controlling for sensory sensitivities. This may suggest that sensory sensitivities are driving the association between neurodivergent traits and ARFID symptoms in our sample of gender diverse adults.

The results of our hierarchical regression analyses suggest there is unique variance associated with sensory sensitivities that is important above and beyond any contributions of autistic or ADHD traits to ARFID symptoms. This is consistent with previous research demonstrating that both autistic and non-autistic gender diverse people experience elevated levels of sensory sensitivities compared to cisgender autistic and non-autistic people, respectively [41]. Although sensory sensitivities may be heightened in gender diverse adults as a result of co-occurring autistic and ADHD traits, this transdiagnostic experience may also uniquely contribute to gender dysphoria. Sensory sensitivities may exacerbate feelings of gender dysphoria by increasing sensitivity to negative sensory experiences of one’s body, such as facial hair growth and menstruation [63, 101]. This heightened distress may in turn facilitate the development and/or maintenance of ARFID symptoms, as a way of managing the negative sensory experiences of one’s body. Given we controlled for shape and weight concerns in our analysis, it is important to consider the potential for multiple pathways from the experience of heightened distress, such as increased drive for muscularity/thinness, as well as to avoid unpleasant sensory experiences. In conclusion, sensory sensitivities may increase the risk for ARFID symptoms in gender diverse adults, over and above the contribution of co-occurring neurodivergent traits.

### Strengths and limitations

By using a dimensional approach, we were able to study associations between ARFID symptoms and sensory sensitivities, autistic, and ADHD traits. This allowed us to account for different types of neurodivergent traits in our model, which is important given the well-established links between sensory sensitivities, autism, and ADHD [76, 89, 102, 103]. In addition, we accounted for weight and shape concerns in our analyses to ensure we were examining restrictive eating behaviours specific to ARFID, rather than those associated with other eating disorders, such as anorexia nervosa [68]. However, it is important to acknowledge the potential limitations of our measures, specifically the AQ-S [72]. The measure includes a social skills subscale which frames impairments in social skills to be characteristic of autism. However, this assumption has been criticised by the autistic community, and many have proposed the double empathy problem as an alternative perspective [104–107]. This framework challenges the deficit model that assumes autistic people are exclusively contributing to difficult or unusual cross-neurotype social interactions. Instead, it emphasises the bidirectionality of social communication and expectations, with both autistic and non-autistic social partners contributing to the outcome. Indeed, research has found autistic people communicate with other autistic people just as effectively as non-autistic people communicate with other non-autistic people [108]. Therefore, it is important to consider the use of current self-report measures of autistic traits and explore alternative tools or adaptations that are informed by the voices of autistic people, encompassing the range of the autistic experience and using accessible and strength-based language.

It is also important to highlight the potential limitations of the GSQ. The GSQ captures sensory processing across seven sensory domains (with six items representing each domain), including items on exteroceptive processing, such as visual and auditory processing, as well as items on proprioception and vestibular processing, which are thought to play a role in interoceptive processing [109]. As sensory sensitivity represents a wide range of distinct sensory domains across both exteroceptive and interoceptive processing, it can be difficult for researchers to precisely classify the type of sensory processing they are investigating, particularly in the context of challenges around classifying sensory processing as purely exteroceptive or interoceptive [110]. The GSQ does not explore distinct subtypes of the sensory experience that might be relevant for research on eating pathology, such as misophonia [111, 112]. Therefore, although the GSQ enables us to capture a number of sensory domains, it does not capture all sensory experiences, and a domain-specific measure would provide greater specificity and internal reliability. In turn, this is likely to result in more nuanced understanding of the role of each sensory processing domain in relation to eating pathology. Lastly, it is important to note that the psychometric properties of the measure may have been impacted by our decision to remove eating-related items due to the overlap between sensory sensitivities and ARFID symptoms.

A limitation of our study is that we did not explore associations between ARFID symptoms and different sensory modalities in our analyses. We chose to focus on the contribution of sensory hyper- and hypo-sensitivities only, as our sample size was moderate, and we did not have the power to explore all sensory domains. It may be the case that certain domains of sensory processing are more relevant to ARFID presentations, as well as co-occurring neurodivergent traits, than others. For example, oral texture sensitivity is the most common sensory difference reported by autistic people [82] and has been found to be an independent predictor of ARFID symptoms in children and adolescents [86]. Hyper-sensitivity to visual stimuli is associated with higher levels of ED symptoms and autistic eating behaviours in autistic adults, while hypo-sensitivity to taste is linked to higher levels of ED symptoms only [113]. Furthermore, we did not explore associations between sensory sensitivities, autistic and ADHD traits, and types of ARFID presentations i.e., aversions to sensory properties, low appetite or limited interest in eating, and/or fear of negative consequences from eating [23]. This is important as drivers of ARFID symptoms may differ depending on the individuals’ neurodivergent and sensory profile, and has implications for clinical support [114]. Further research is needed to explore these relations with larger samples to enable a more fine-grained and nuanced approach. Lastly, our cross-sectional design does not allow us to establish causality or directionality with respect to the associations between ARFID symptoms, sensory sensitivities, and neurodivergent traits.

A strength of our study is the recruitment of a moderate sample of gender diverse people who identified with a range of diverse gender identities. For example, previous research has reported challenges recruiting non-binary participants [35], whereas just under 50% of our sample identified as non-binary (including trans masculine and trans feminine non-binary identities). However, the small sample sizes for each gender diverse identity in our study limited our ability to conduct regression analyses for each group separately. This is an important limitation given previous research has found ED prevalence rates and presentations to differ across gender diverse groups [11, 13]. For example, transmasculine people assigned female at birth often report engaging in weight control behaviours and excessive exercise to achieve a more lean and muscular body and appear less feminine [115–117]. For transfeminine people assigned male at birth, engagement in weight control behaviours may be used to appear more traditionally feminine, such as altering body shape to align with feminine body ideals and reducing facial and body hair growth [7, 118–120]. Furthermore, studies exploring eating pathology in non-binary people have found thinness to be driven in part by a desire for androgynous gender expression [121–123]. Although these findings are specific to eating pathology related to weight and shape concerns rather than ARFID, further support for exploring gender diverse groups separately is reported in autism research. For example, higher levels of autistic traits have been found in non-binary and gender expansive individuals compared to binary transgender adults [42]. Therefore, neurodivergent traits may interact with gender identity to influence ARFID symptom development and presentation.

Although we controlled for sex assigned at birth in our regression analyses, gender diverse people assigned female at birth were overrepresented in our sample, even when considering recent gender clinic referral data [124]. This appears to be a challenge shared by other ARFID studies with gender diverse people, as Zickgraf, Garwood [35] report just over 18% of their sample were assigned male at birth. In their study, gender diverse people assigned female at birth were reported to score significantly higher on the NIAS compared to those assigned male at birth [35]. However, given the underrepresentation of gender diverse people assigned male at birth, future research is needed to examine the intersection of sex assigned at birth, gender identity, and ARFID symptoms. Further, the current sample is predominantly White and educated, highlighting the need to consider a range of recruitment strategies to reach communities who may be less familiar with or comfortable engaging in research.

It is important to acknowledge the high levels of neurodivergence and mental health diagnoses reported by our sample. Although previous research has reported gender diverse people to be more likely to be autistic and/or have ADHD, just under 30% of our sample reported receiving an autism diagnosis, whilst 25% reported an ADHD diagnosis. These figures are elevated compared to rates reported by other studies with gender diverse samples and may have influenced the relations between autistic traits, ADHD traits, sensory sensitivity, and ARFID symptoms in our study. In a recent meta-analysis, Kallitsounaki and Williams [45] found autism diagnoses to have a pooled estimate of 11% in gender diverse people. A similar rate of 10% has been reported for ADHD diagnoses in gender diverse people [125]. However, the authors acknowledge large variations in prevalence rates across studies, potentially due to differences in study designs (e.g., clinical studies vs. population studies) and participant type (e.g., individuals who have received a gender dysphoria/gender incongruence diagnosis vs. individuals who report gender incongruence). Prevalence estimates of autism diagnoses are lower in studies recruiting individuals from gender clinics who have received a diagnosis of gender dysphoria compared to individuals from the general population who identify as gender diverse [45]. Although this could suggest overestimation of self-reported neurodivergent diagnoses in individuals recruited from the community, it is also possible that neurodivergent diagnoses are under-diagnosed in gender diverse people attending gender clinics [45], or may not be explored or identified until later on in their gender transition. In addition, our recruitment materials explicitly stated that we were investigating neurodivergent characteristics alongside eating behaviours and gender diversity. This may have attracted more people with neurodivergent diagnoses or self-identified neurodivergent characteristics than would typically be expected in a gender diverse sample. In addition to high rates of neurodivergent diagnoses in our sample, around 75% of our sample reported a co-occurring mental health diagnosis, excluding eating disorders. Although previous studies with gender diverse people also report high levels of mental health diagnoses [125–127], this may be a potential confounding factor due to the high co-occurrence between eating disorders and mental health difficulties, including anxiety and depression [15, 128–130].

### Clinical implications

The findings from our study have implications for clinicians’ understanding of ARFID presentations in gender diverse people, as well as considerations for treatment in this group. Firstly, clinicians supporting gender diverse people should be aware of the strong positive association between sensory sensitivities and ARFID symptoms. This may indicate a transdiagnostic feature that is elevated in neurodivergent individuals, and so clinicians should carefully assess this when working with individuals with ARFID, using standardised questionnaires where appropriate. Clinicians should aim to establish a shared understanding of sensory needs with the gender diverse individual they are supporting, as well as how these needs may intersect with eating difficulties. Gender diverse people may experience a complex intersection of gender-related distress, sensory sensitivities, and eating behaviours. The individual’s experience of neurodivergence, whether this relates to traits, identity or a formal diagnosis, are likely to factor into their experience of sensory sensitivities and ARFID and should also be carefully considered. It is important that clinicians aim to disentangle and understand these experiences to reach an accurate shared formulation of eating problems.

In conclusion, our findings demonstrate higher levels of ARFID symptoms are associated with higher levels of autistic traits, ADHD traits, and sensory sensitivities in a gender diverse sample. Furthermore, sensory sensitivities, specifically hyper-sensitivity, uniquely predicted levels of ARFID symptoms once we accounted for autistic and ADHD traits. This pattern of findings suggests sensory hyper-sensitivities may be particularly relevant to ARFID symptomatology in gender diverse adults, compared to other traits that are relevant to neurodivergence. Future research is needed with larger and more diverse samples to corroborate and extend these findings.

## Electronic supplementary material

Below is the link to the electronic supplementary material.

Supplementary Material 1

## Data Availability

Data are available at https://osf.io/s4eua/.
