# Supplementary material for: Avoidant/restrictive food intake disorder (ARFID) symptoms in gender diverse adults and their relation to autistic traits, ADHD traits, and sensory sensitivities
Source: J Eat Disord. 2025 Feb 17;13:33. doi: 10.1186/s40337-025-01215-z (PMC11834292; doi:10.1186/s40337-025-01215-z)
Supplement: Supplementary file 1 — Supplementary Material 1 [file 40337_2025_1215_MOESM1_ESM.docx]

**Supplementary Materials**

**Table S1.**

*Additional Sample Characteristics (N = 182).*

|  | Frequency | % of the sample |
| --- | --- | --- |
| **Ethnicity** |  |  |
| White | 163 | 89.6 |
| Mixed or multiple ethnic groups | 10 | 5.5 |
| Asian or Asian British | 3 | 1.6 |
| Black, Black British, Caribbean or African | 2 | 1.1 |
| Other ethnic group | 2 | 1.1 |
| Prefer not to say/Missing | 2 | 1.1 |
| **Highest level of qualification**^a^ |  |  |
| No formal qualifications | 1 | 0.5 |
| GCSE passes, apprenticeships, or equivalent | 21 | 11.5 |
| A levels or equivalent | 49 | 26.9 |
| Bachelor’s degree, Higher National Certificate/Diploma | 65 | 35.7 |
| Master’s level and above | 43 | 23.6 |
| Prefer not to say | 3 | 1.6 |
| **Sexual orientation** |  |  |
| Straight or heterosexual | 4 | 2.2 |
| Gay or lesbian | 28 | 15.4 |
| Bisexual | 48 | 26.4 |
| Pansexual | 27 | 14.8 |
| Asexual | 48 | 26.4 |
| Other sexual orientation | 25 | 13.7 |
| Prefer not to say/Missing | 2 | 1.1 |

*Note*. ^a^Eight categories were collapsed into six for presentation in this table.

**Table S2.**

*Descriptive statistics by gender identity.*

|  | Trans masculine  (N= 62) | Trans feminine  (N= 28) | Non-binary  (N= 64) | Gender expansive  (N= 28) |
| --- | --- | --- | --- | --- |
|  | Mean (SD) | Mean (SD) | Mean (SD) | Mean (SD) |
| ARFID symptoms (NIAS) | 14.97 (9.88) | 17.14 (12.54) | 14.88 (10.79) | 19.11 (8.43) |
| Picky eating | 6.15 (5.01) | 7.64 (5.09) | 6.20 (4.89) | 8.21 (3.87) |
| Appetite | 5.34 (4.22) | 5.39 (5.18) | 5.59 (4.29) | 7.00 (4.48) |
| Fear | 3.48 (4.29) | 4.11 (5.06) | 3.09 (4.24) | 3.89 (3.97) |
| Autistic traits (AQ-S) | 77.54 (13.39) | 80.21 (11.48) | 81.18 (11.69) | 82.71 (11.19) |
| Social skills | 21.97 (4.85) | 23.96 (4.65) | 24.72 (3.80) | 24.50 (4.33) |
| Routine | 12.74 (2.41) | 13.07 (2.31) | 13.11 (2.22) | 13.18 (1.98) |
| Switching | 12.65 (2.28) | 13.13 (2.10) | 13.19 (2.40) | 13.21 (2.02) |
| Imagination | 19.94 (5.36) | 19.40 (4.16) | 19.86 (5.73) | 19.79 (5.14) |
| Numbers and patterns | 13.91 (3.48) | 14.29 (3.82) | 13.99 (3.53) | 15.79 (3.19) |
| ADHD traits (ASRS) | 12.19 (4.58) | 11.75 (4.29) | 12.09 (3.68) | 13.96 (2.65) |
| Inattentive | 5.77 (2.36) | 6.04 (1.88) | 5.95 (1.81) | 6.82 (1.31) |
| Hyperactivity/impulsivity motor | 3.48 (1.61) | 3.04 (1.71) | 3.36 (1.41) | 3.75 (1.00) |
| Hyperactivity/impulsivity verbal | 2.31 (1.29) | 1.82 (1.52) | 2.09 (1.35) | 2.68 (1.19) |
| Sensory sensitivities (GSQ)* | 66.43 (23.81) | 66.44 (23.83) | 69.41 (19.67) | 78.14 (16.43) |
| Hyper-sensitivity | 36.12 (14.03) | 36.04 (12.24) | 38.47 (11.50) | 42.73 (10.38) |
| Hypo-sensitivity | 30.31 (11.11) | 30.39 (13.05) | 30.94 (9.66) | 35.42 (7.59) |
| Shape concern (EDE-Q) | 3.65 (1.57) | 3.66 (1.76) | 3.54 (1.46) | 3.16 (1.56) |
| Weight concern (EDE-Q) | 3.24 (1.67) | 3.31 (1.82) | 3.05 (1.49) | 2.77 (1.51) |

Note: NIAS: Nine-Item ARFID Scale; ARFID: Avoidant/Restrictive Food Intake Disorder; AQ-S: Autism Spectrum Quotient – Short; ASRS: Adult ADHD Self-Report Scale; GSQ: Glasgow Sensory Questionnaire; EDE-Q: Eating Disorder Examination Questionnaire.

Trans masculine group includes those identifying as trans masculine and non-binary trans masculine. Trans feminine group includes those identifying as trans feminine and non-binary trans feminine.

**Table S3.**

*Spearman’s correlations between neurodivergent trait subscales, sensory sensitivities, and NIAS total score for transmasculine participants (N = 62).*

|  | NIAS | GSQ Hyper-sensitivity | GSQ Hypo-sensitivity |
| --- | --- | --- | --- |
| ARFID symptoms (NIAS) |  | .589*** | .396** |
| Autistic traits (AQ-S) |  |  |  |
| Social skills | .393** | .550*** | .302* |
| Routine | .407** | .583*** | .479*** |
| Switching | .293* | .461*** | .454*** |
| Imagination | .329** | .444*** | .254* |
| Numbers and patterns | .328** | .555*** | .542*** |
| ADHD traits (ASRS) |  |  |  |
| Inattentive | .141 | .276* | .490*** |
| Hyperactivity/impulsivity motor | .249 | .423*** | .569*** |
| Hyperactivity/impulsivity verbal | .234 | .263* | .338** |

**p<.05, **p<.01, ***p<.001*

Note: NIAS: Nine-Item ARFID Scale; ARFID: Avoidant/Restrictive Food Intake Disorder; AQ-S: Autism Spectrum Quotient – Short; ASRS: Adult ADHD Self-Report Scale; GSQ: Glasgow Sensory Questionnaire.

**Table S4.**

*Spearman’s correlations between neurodivergent trait subscales, sensory sensitivities, and NIAS total score for transfeminine participants (N = 28).*

|  | NIAS | GSQ Hyper-sensitivity | GSQ Hypo-sensitivity |
| --- | --- | --- | --- |
| ARFID symptoms (NIAS) |  | .591*** | .324 |
| Autistic traits (AQ-S) |  |  |  |
| Social skills | .528** | .610*** | .333 |
| Routine | .322 | .238 | -.095 |
| Switching | .113 | .399* | .068 |
| Imagination | .257 | .224 | .236 |
| Numbers and patterns | .277 | .509** | .660*** |
| ADHD traits (ASRS) |  |  |  |
| Inattentive | -.008 | .399* | .487** |
| Hyperactivity/impulsivity motor | .284 | .515** | .617*** |
| Hyperactivity/impulsivity verbal | -.159 | .235 | .539** |

**p<.05, **p<.01, ***p<.001*

Note: NIAS: Nine-Item ARFID Scale; ARFID: Avoidant/Restrictive Food Intake Disorder; AQ-S: Autism Spectrum Quotient – Short; ASRS: Adult ADHD Self-Report Scale; GSQ: Glasgow Sensory Questionnaire.

**Table S5.**

*Spearman’s correlations between neurodivergent trait subscales, sensory sensitivities, and NIAS total score for non-binary participants (N = 64).*

|  | NIAS | GSQ Hyper-sensitivity | GSQ Hypo-sensitivity |
| --- | --- | --- | --- |
| ARFID symptoms (NIAS) |  | .530*** | .527*** |
| Autistic traits (AQ-S) |  |  |  |
| Social skills | .126 | .343** | .104 |
| Routine | .163 | .388** | .087 |
| Switching | -.115 | .183 | -.016 |
| Imagination | .238 | .435*** | .217 |
| Numbers and patterns | .339** | .594*** | .427*** |
| ADHD traits (ASRS) |  |  |  |
| Inattentive | .272* | .300* | .427*** |
| Hyperactivity/impulsivity motor | .332** | .451*** | .612*** |
| Hyperactivity/impulsivity verbal | .170 | .261* | .376** |

**p<.05, **p<.01, ***p<.001*

Note: NIAS: Nine-Item ARFID Scale; ARFID: Avoidant/Restrictive Food Intake Disorder; AQ-S: Autism Spectrum Quotient – Short; ASRS: Adult ADHD Self-Report Scale; GSQ: Glasgow Sensory Questionnaire.

**Table S6.**

*Spearman’s correlations between neurodivergent trait subscales, sensory sensitivities, and NIAS total score for gender expansive participants (N = 28).*

|  | NIAS | GSQ Hyper-sensitivity | GSQ Hypo-sensitivity |
| --- | --- | --- | --- |
| ARFID symptoms (NIAS) |  | .366 | .118 |
| Autistic traits (AQ-S) |  |  |  |
| Social skills | .003 | .581** | .385* |
| Routine | .116 | .262 | .186 |
| Switching | .291 | .420* | .292 |
| Imagination | .517** | .290 | .225 |
| Numbers and patterns | .129 | .329 | .445* |
| ADHD traits (ASRS) |  |  |  |
| Inattentive | -.036 | .042 | .298 |
| Hyperactivity/impulsivity motor | -.109 | .305 | .608*** |
| Hyperactivity/impulsivity verbal | -.279 | -.017 | .324 |

**p<.05, **p<.01, ***p<.001*

Note: NIAS: Nine-Item ARFID Scale; ARFID: Avoidant/Restrictive Food Intake Disorder; AQ-S: Autism Spectrum Quotient – Short; ASRS: Adult ADHD Self-Report Scale; GSQ: Glasgow Sensory Questionnaire.
